# Noxious radiant heat evokes bi-component nociceptive withdrawal reflexes in spinal cord injured humans—A clinical tool to study neuroplastic changes of spinal neural circuits

**DOI:** 10.3389/fnhum.2023.1141690

**Published:** 2023-05-02

**Authors:** Steffen Franz, Laura Heutehaus, Anke Tappe-Theodor, Norbert Weidner, Rolf-Detlef Treede, Sigrid Schuh-Hofer

**Affiliations:** ^1^Spinal Cord Injury Center, Heidelberg University Hospital, Heidelberg, Germany; ^2^Department of Molecular Pharmacology, Medical Faculty Heidelberg, Institute of Pharmacology, Heidelberg University, Heidelberg, Germany; ^3^Department of Neurophysiology, Mannheim Center for Translational Neuroscience, Medical Faculty Mannheim, Ruprecht-Karls-University of Heidelberg, Mannheim, Germany; ^4^Department of Neurology and Epileptology, University of Tübingen, Tübingen, Germany

**Keywords:** spinal cord injury, neuroplastic changes, neuropathic pain, spasticity, hyperexcitability, maladaptive plasticity, withdrawal reflexes

## Abstract

Investigating nocifensive withdrawal reflexes as potential surrogate marker for the spinal excitation level may widen the understanding of maladaptive nociceptive processing after spinal cord injury (SCI). The aim of this prospective, explorative cross-sectional observational study was to investigate the response behavior of individuals with SCI to noxious radiant heat (laser) stimuli and to assess its relation to spasticity and neuropathic pain, two clinical consequences of spinal hyperexcitability/spinal disinhibition. Laser stimuli were applied at the sole and dorsum of the foot and below the fibula head. Corresponding reflexes were electromyography (EMG) recorded ipsilateral. Motor responses to laser stimuli were analyzed and related to clinical readouts (severity of injury/spasticity/pain), using established clinical assessment tools. Twenty-seven participants, 15 with SCI (age 18–63; 6.5 years post-injury; AIS-A through D) and 12 non-disabled controls, [non-disabled controls (NDC); age 19–63] were included. The percentage of individuals with SCI responding to stimuli (70–77%; *p* < 0.001), their response rates (16–21%; *p* < 0.05) and their reflex magnitude (*p* < 0.05) were significantly higher compared to NDC. SCI-related reflexes clustered in two time-windows, indicating involvement of both A-delta- and C-fibers. Spasticity was associated with facilitated reflexes in SCI (Kendall-tau-b *p* ≤ 0.05) and inversely associated with the occurrence/severity of neuropathic pain (Fisher’s exact *p* < 0.05; Eta-coefficient *p* < 0.05). However, neuropathic pain was not related to reflex behavior. Altogether, we found a bi-component motor hyperresponsiveness of SCI to noxious heat, which correlated with spasticity, but not neuropathic pain. Laser-evoked withdrawal reflexes may become a suitable outcome parameter to explore maladaptive spinal circuitries in SCI and to assess the effect of targeted treatment strategies. Registration: https://drks.de/search/de/trial/DRKS00006779.

## 1. Introduction

Beyond the loss of sensorimotor function, spinal cord injury (SCI) entails numerous immediate and indirect debilitating consequences, such as spasticity and central neuropathic pain (Weidner et al., 2017). Indeed, most of the individuals with SCI develop at least one of these complications. The occurrence of spasticity is known to be closely related to hyperexcitability and consecutive hyperreflexia (Adams and Hicks, 2005). Thus, well-established, and overall effective therapeutic approaches, such as the administration of baclofen, aim to attenuate neuronal excitability, for instance by enhancing pre-synaptic inhibition (de Sousa et al., 2022). Existing treatment options for SCI-related neuropathic pain are also based on the assumption of underlying neural hyperexcitability and therefore aim toward similar directions, albeit with a mostly less vigorous effect (Finnerup, 2017). The reasons for this discrepancy remain unclear, but may be related, for instance, to divergent underlying mechanisms in the development of these detrimental sequelae of SCI.

Thus, aiming at improved and more effective therapeutic strategies, a better understanding of underlying pathophysiological processes after SCI is essential. Complex maladaptive processes—both at the spinal and supraspinal level—are discussed to contribute to SCI-related neuropathic pain (Finnerup, 2017; Sliwinski et al., 2018; Zhang et al., 2021). Among other mechanisms, enhancement of neuronal excitability and/or disinhibition of (inter-)neuronal networks—as sequelae of aberrant (structural and functional) plasticity—are assumed to represent relevant factors in this context (Costigan et al., 2009; Kuner, 2010; Gómez-Soriano et al., 2016).

In contrast to experimental models, which enable to investigate spinal hyperexcitability by selectively exploring excitatory and inhibitory neural circuitries at the cellular level, the reliable assessment of the spinal excitation level in humans is challenging. Yet, one reasonable approach is exploring the neurophysiological reflex behavior, as such reflexes in principle reflect the spinal excitation level based on distinct readouts such as response rates and magnitudes (Sandrini et al., 2005). Reflex studies may either apply innocuous stimuli, e.g., to study the impact of SCI on the H-reflex (Lee et al., 2005; Knikou, 2008; Gómez-Soriano et al., 2018; Pion et al., 2021), or noxious stimuli to assess the effect of injured sensorimotor pathways on nociceptive withdrawal—also referred to as nocifensive—reflexes (Lim et al., 2011; Linde et al., 2021). Previous clinical and preclinical studies on the effect of SCI on nocifensive reflexes revealed motor hyperresponsiveness and enlarged reflex receptive fields (RRF) (Dimitrijevic and Nathan, 1968; Andersen et al., 2004; Biurrun Manresa et al., 2014). However, like in most human studies on nocifensive reflexes (Shahani and Young, 1971; Meinck et al., 1981; Ellrich and Treede, 1998; Andersen et al., 1999; Sonnenborg et al., 2001; Sandrini et al., 2005), investigations on the reflex behavior of individuals with SCI used suprathreshold electrical (Kugelberg et al., 1960; Grimby, 1963; Andersen et al., 2004; Biurrun Manresa et al., 2014) rather than thermal stimuli (Willer et al., 1979; Mørch et al., 2007) to evoke withdrawal reflexes. While electrical stimulation paradigms typically activate non-selectively both large and small primary afferents (Sandrini et al., 2005), the use of radiant heat stimuli enables to more selectively stimulate thinly-myelinated A-delta fibers and unmyelinated C-fibers (Bromm et al., 1983; Bromm and Treede, 1987; Magerl et al., 1999).

In human pain research, laser stimuli are widely used to test the integrity of A-delta and C-fiber function in individuals with neuropathic pain and explore supraspinal nociceptive processing (Treede et al., 2003; Fabry et al., 2020).

Backed on this, and with the goal of a proof of concept, our primary aim was to investigate the laser-evoked reflex behavior of individuals with SCI. We hypothesized that individuals with SCI would show hyperresponsiveness to noxious radiant heat (laser stimuli) as a result of hyperexcitability compared with non-disabled controls (NDC). Provided that individuals with SCI are indeed characterized by more pronounced motor responses, we also intended to conduct subsequent investigations on a possible association between spasticity (presence and severity), neuropathic pain (presence and intensity), and reflex hyperresponsiveness as related indications of hyperexcitability.

## 2. Materials and methods

This prospective duo-centric cross-sectional explorative observational study was designed and conducted at the SCI Center Heidelberg (Heidelberg University Hospital, Germany) and the Department for Neurophysiology, Ruprecht-Karls-University of Heidelberg (MCTN–Mannheim Center for Translational Neuroscience, Medical Faculty Mannheim) as part of a clinical observational study focusing on chronic central neuropathic pain after SCI. It was conducted in accordance with the Declaration of Helsinki and approved by the local ethics committees of the Medical Faculties of Heidelberg and Mannheim, Germany (S-660/2013 and 603N/2014). It was registered at the German Clinical Trials Registry (DRKS00006779). All participants signed informed consent prior to study enrolment.

### 2.1. Participants and recruitment

All individuals with SCI were recruited by convenience sampling among in- and out-patients of the SCI center and by sifting the list of participants in the European Multicenter Study about SCI (EMSCI) at the Heidelberg study site (Curt, 2005). Inclusion required full legal age. The age limit was 65 years. Ability to give informed consent was a prerequisite for inclusion. Individuals with SCI had to be in the chronic stage based on the EMSCI schedule (time frame 300–546 days after injury, pursuant to the exam stage 12 months)^1^ and should present with a neurological level of injury (NLI) between C3 and T10, provided that unimpaired spontaneous respiration was warranted. Potentially interfering medication such as pain medication or spasmolytics were documented.

To relate findings in individuals with SCI to those of individuals without SCI, age and gender-matched NDC were included in this study, recruited among the clinical staff of the study sites.

### 2.2. Clinical examination and classification of individuals with spinal cord injury

Participants with SCI were neurologically examined and classified according to the 7th edition of the International Standards for Neurological Classification of SCI (ISNCSCI) by expertly trained assessors (Kirshblum et al., 2011; Schuld et al., 2013; Franz et al., 2022).

Spasticity was assessed by using both the SCAT (spinal cord assessment tool for spastic reflexes) (Benz et al., 2005) and the Modified Ashworth Scale (MAS) (Bohannon and Smith, 1987). According to common clinical practice, a grading of ≤ 1 for either of the assessments was not considered clinically relevant as it does not commonly represent an indication for treatment. Thus, clinically relevant spasticity was only assumed if at least one of the joints exhibited a grade of > 1 for either MAS or SCAT. For statistical analyzes mean values of all relevant sub-scores (i.e., examined joints) were used.

Central (below-level) neuropathic pain was assessed according to current guidelines (Treede et al., 2008; Finnerup, 2013; Widerström-Noga et al., 2016) including the SCI pain instrument (SCIPI) as screening tool (Franz et al., 2017). All individuals presenting with at-level neuropathic pain were excluded from this analysis. Routinely assessed magnetic resonance imaging of the spine were evaluated to exclude misattribution of pain types (e.g., cauda equina syndrome versus spinal cord lesion to distinguish between at- and below-level neuropathic pain) and to exclude macroscopic disruption of spinal cord continuity. The final assignment of participants was referred to as clinical neuropathic pain grading (CPG). Additionally, the numeric rating scale (NRS) was used for each participant on the day of the study as a widely established instrument for assessing spontaneous pain intensity, specifically referring to the entity of pain perceived as most severe by the participant in each case (Table 1). According to common practice, a result on the NRS of ≥ 4 was considered clinically relevant (Karcioglu et al., 2018).

**TABLE 1:** Demographics and basic clinical characteristics of participants with spinal cord injury.

ID	Age	Gender	AIS	NLI	TSI	SCIPI	CPG	NocP	NRS	MAS	SCAT	L-NRS FS	L-NRS FD	L-NRS FH
	Years		Range A–E		Years	Range 0–7	Yes/No	Yes/No	Range 0–10	Range 0–5	Range 0–3	Range 0–100	Range 0–100	Range 0–100
01	53	M	A	C2	32.4	2	N	Y	6	2.67	1.00	0	0	0
02	31	M	A	T8	13.3	0	N	N	0	2.33	1.17	0	0	0
03	36	M	A	T4	14.4	1	N	N	0	4.17	1.33	0	0	0
04	45	F	A	T4	2.7	7	Y	N	5	0.17	0.00	0	0	0
05	62	M	A	T4	6.1	5	Y	N	7	0.33	0.00	0	0	n.a.^a^
06	42	F	A	T7	1.2	7	Y	N	7	0.67	0.67	0	0	0
07	18	F	D	T5	1.1	1	N	Y	1	3.67	0.83	2	6	2
08	59	M	D	C6	1.5	2	N	Y	1	2.17	0.83	2	4	2
09	26	F	C	T3	1.7	4	Y	N	7	4.33	1.17	3	7	3
10	62	F	D	C3	3.8	6	Y	N	7	2.16	0.50	11	11	11
11	63	M	D	T5	8.3	3	N	Y	3	MD^b^	1.67	n.a.^a^	1	n.a.^a^
12	61	F	D	T5	1.9	3	Y	N	4	0.00	0.00	10	9	n.a.^a^
13	41	F	D	C5	0.9	5	N	Y	6	MD^b^	0.50	n.a.^a^	25	n.a.^a^
14	46	M	D	C2	2.0	4	Y	N	8	0.83	0.17	1	0	n.a.^a^
15	54	M	D	T1	6.7	5	Y	N	6	0.33	0.16	5	4	5
Mean	46.6	–	–	–	6.5	3.7	–	–	4.5	1.99	0.67	–	–	–
± SD	13.8	–	–	–	8.1	2.1	–	–	2.7	1.46	0.52	–	–	–

^a^Not available, either not performed or for technical reasons. ^b^Missing data, no complete assessment. Data on CPG/NocP and NRS are directly related to each other. In the case of CPG = N as well as NocP = N and NRS > 0, the pain entity would be neither neuropathic nor nociceptive, but according to the applicable classification, other/unknown pain (Bryce et al., 2012a). AIS, ASIA impairment scale; ASIA, American Spinal Injury Association; C, cervical; CPG, clinical pain grading of central neuropathic pain; F, female; FD, dorsum of the foot; FS, sole of the foot; FH, head of the fibula; L-NRS, laser stimulus painfulness numeric rating scale; M, male; MAS, Modified Ashworth Scale; MD, missing data; N, no; n.a., not available; NocP, nociceptive pain; NRS, numeric rating scale (clinically meaningful ≥ 4, boldface); SCAT, spinal cord assessment tool for spastic reflexes; SCIPI, spinal cord injury pain instrument (indicative of potential NeuP ≥ 4/7); SD, standard deviation; T, thoracic; TSI, time since injury; Y, yes.

### 2.3. Withdrawal reflexes elicited from laser stimulation

Since this was an exploratory study, care was taken in the present protocol to ensure the greatest possible difference between individuals with SCI and NDC. This could be achieved by using stimulation intensities that–according to previous experience–would hardly elicit a reflex activity in NDC but would in SCI. The laser energies used (see below) have long been established and proven at the study site to (1) elicit stable laser-evoked potentials, (2) be safe/harmless with respect to possible skin irritation/injury, and (3) elicit little reflex behavior in healthy subjects. Indeed, previous publications have shown that similar laser energies are effective in activating nociceptors without eliciting reflex activity (Lenz et al., 1998; Treede et al., 2003; Vogel et al., 2003; Mørch et al., 2007).

To elicit laser-induced reflexes, the sole of the foot (FS) (Kugelberg et al., 1960; Grimby, 1963; Andersen et al., 1999, 2001, 2004; Sonnenborg et al., 2001; Biurrun Manresa et al., 2014) and the dorsum of the foot (FD) (Devigili et al., 2008; Haanpää et al., 2011; Di Stefano et al., 2017) were stimulated to assess withdrawal reflexes. Since we aimed to estimate the conduction velocity of the fiber types involved, additional stimuli were applied below the head of the fibula (FH) about 400 mm proximal to FD in a subgroup of SCI individuals (*n* = 10).

**FIGURE 1:**
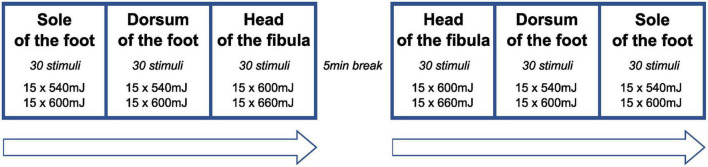
Sequence of stimulation series. At each stimulation site, the two stimulation energies were applied in a random sequence (inter-stimulus interval: 7,000–9,000 ms) to avoid habituation of reflex behavior. At the head of the fibula, laser stimulation was only performed in a subset of participants (*n* = 10).

The stimulation protocol is illustrated in Figure 1. Lower stimulation intensities (540, 600 mJ) were applied to FH and FD compared to FS (600, 660 mJ) because hairy skin (FH, FD) and glabrous skin (FS) are known to differ in their heat response properties (Taylor et al., 1993; Treede et al., 1995; Granovsky et al., 2005). Following previous protocols (Sandrini et al., 2005), reflex activity was recorded from the anterior tibialis muscle (TA) and soleus muscle (SO). Bipolar surface electromyography (EMG) electrodes were placed on the TA (reference electrode: patella) and SO (reference electrode: lateral malleolus) ipsilateral to the stimulation side. Visual inspection and analysis of EMG recordings were done using the BrainAmp ExG-amplifier (16 channels) for bipolar EMG recording (Brain Products, Gilching, Germany) and Brain Vision Analyzer Software (Version 2.1, Brain Products, Gilching, Germany). Radiant heat stimuli were generated by a thulium laser (wavelength 2 μm, beam diameter 5 mm, 1 ms stimulus duration; Themis, Starnberg, Germany). The stimulation series consisted of 60 laser stimuli. Both for safety reasons (to avoid skin damage) and to prevent receptor fatigue or sensitization, the laser beam was slightly moved after each stimulation within the stimulation site (Greffrath et al., 2007). Further strategies to minimize the likelihood of habituation to the laser stimuli included the random application of three different inter-stimulus intervals (6,000–7,000–9,000 ms) (von Dincklage et al., 2013) and the random application of two different laser intensities (see above) within each stimulation series.

Three seconds after applying the stimulus, an acoustic signal was provided, prompting the participants to rate the intensity of the perceived stimulus (NRS = 0: no painful sensation, NRS = 100: worst imaginable pain). Study participants and the investigator wore goggles to avoid eye damage. After each stimulus, the beam was slightly moved to avoid peripheral fatigue or sensitization.

Participants were seated in a deckchair with a movable backrest. Hands were resting on armrests and legs were deflected at an angle of ∼150° in a temperature-controlled environment at 22°C. The skin temperature was recorded (VisioFocus 06400, Tecnimed, Italy). If the skin temperature was below 32°C, physical measures (hot water bath) were applied. Before starting the experiment, correct channel recording was ensured by triggering the Achilles tendon reflex and evaluating the EMG activity induced at the SO. If possible, correct channel recording of the TA was checked by recording EMG activity in dorsiflexion of the foot.

Electromyography (EMG) data were band-pass filtered (1–450 Hz), segmented (from 500 ms pre-stimulus to 3,000 ms post-stimulus), rectified (sampling rate: 1,000 Hz). A reflex was defined by polyphasic EMG activity exceeding two standard deviations above background noise. Latencies of each motor response were determined using Brain Vision Analyzer Software. The toolbox of the Systat Software Sigma Plot (Version 14) was applied for area under the curve (AUC)-analysis.

The following outcome parameters were assessed: (1) reflex latencies (ms) for temporal pattern evaluation of the reflex behavior, (2) proportion of participants displaying any reflex activity (“responders”), (3) individual percentage of motor responses to a sequence of 60 laser stimuli (“response rate”), (4) magnitude of responses as measured by AUC in relation to reflex muscle activity (surface EMG signals), (5) pain ratings of applied laser stimuli.

### 2.4. Statistical analysis

SPSS was used for statistical analysis (IBM SPSS Statistics 20). Since data were not normally distributed, non-parametric tests were applied.

For basic evaluation of the overall prevalence of reflex activity, study participants were first categorized as “responders” and “non-responders” based on any recorded reflex activity, regardless of the stimulation or recording site. However, since the reflex activity of individuals with SCI occurred in two different time-windows (TW, see below), further analysis of reflex activity also considered the (respective) TW of their occurrence. Subsequently, the “response rates” of the study participants to a series of 60 stimuli were examined. The results were analyzed separately for both study groups (SCI and NDC), depending on the three stimulation sites and the TW of their occurrence. Wilcoxon rank-sum test was used to evaluate differences by group comparison (NDC versus SCI). Based on our stated *a priori* hypotheses [noxious radiant (laser) reflex hyperresponsiveness in SCI], comparative statistical analyses on “responder” and “response rates” between NDC and SCI, as well as subsequent correlation analyses between spasticity and reflex behavior were one-tailed (Dimitrijevic and Nathan, 1968; Shahani and Young, 1971). All other results are based on two-tailed tests. Statistical tests were selected based on the level of measurement of the parameters to be tested for associations (spasticity, neuropathic pain, and reflex response rates). Fisher’s exact test was performed to test the association (contingency) between CPG and clinically relevant spasticity (binary data = yes/no). The Eta-coefficient was applied to assess CPG’s association with the severity of spasticity (MAS/SCAT) and reflex response rates (binary and metric data). Kendall rank correlation (Kendall-tau-b) coefficient was used to test associations of the intensity of neuropathic pain and the severity of spasticity (SCAT/MAS/NRS) with response rates, as well as the severity of spasticity (MAS/SCAT) with the intensity (NRS) of neuropathic pain (metric data). Where appropriate, the corresponding effect sizes were provided: for Wilcoxon rank-sum test *r* < 0.3 = small; *r* < 0.5 = medium, *r* > 0.5 = large; for Fisher’s exact test phi/Φ < 0.3 = small; Φ 0.3–0.5 = medium; Φ > 0.5 = large (Cohen, 1992). The significance level was set to ≤ 0.05.

## 3. Results

Fifteen individuals with SCI (age 18–63; 7x female) were enrolled. Due to ongoing recruitment challenges, two individuals with SCI and an NLI of C2 were also included after demonstrating unremarkable spontaneous respiration (Table 1). Most of the participants presented with motor incomplete SCI according to the ASIA impairment scale (AIS: 1x AIS-C, 8x AIS-D) (Kirshblum et al., 2011). The remaining 6 individuals presented with a sensory and motor complete SCI (AIS-A). Time since injury (TSI) varied across the cohort (6.5 ± 8.4 years; range: 0.91–32.4). Clinically relevant spasticity (MAS/SCAT > 1 in any joint) was examined in 47% of the SCI-cohort (7/15), two of which also presented clinically relevant below-level neuropathic pain. The presence of spasticity was independent from the injury severity according to AIS (Fisher’s exact *p* > 0.05, Φ = 0.06). For one individual with SCI the MAS was not completely assessed leading to missing data.

Clinically relevant pain severity (NRS ≥ 4)—irrespective of its entity (neuropathic versus others)—was reported by ten participants (NRS 6.1 ± 1.1), eight of which were diagnosed with below-level neuropathic pain according to CPG (NRS 6.4 ± 1.3). In detail, the 7-item SCIPI screening for neuropathic pain was positive in 53% of participants (8/15). In two participants, the initial SCIPI-screening was overruled by the clinical assessment (ID 12 and 13; Table 1). Three of the participants with SCI and below-level neuropathic pain used relevant agents (ID 4, 12 and 14 either pregabalin or gabapentin). Seven persons with SCI stated to use spasmolytic medication (only baclofen, ID 1, 2, 5, 7, 9, 10, and 11), six of which were characterized by clinically relevant spasticity (Table 1). In the NDC-cohort, 12 age and gender matched participants (age: 19–63; 6x females) were examined. None of them reported spontaneous pain of any entity.

### 3.1. Nocifensive reflex activity in comparison between SCI and non-disabled controls

Upon completion of the experiments, evidence of two major subgroups in both SCI and NDC emerged. One subgroup displayed recordable withdrawal reflexes (“responders”), whereas the other group (“non-responders”) did not. Due to technical issues, EMG recordings after foot sole stimulation are missing in two participants of the SCI-cohort. Among the “responders,” withdrawal reflexes were characterized by activity in potentially two different time-windows (TW1 and TW2); (see also below: “temporal pattern of reflexes”). As *a priori* expected, the reflex behavior in the NDC was found to be less frequent. In particular, in TW1, NDC did not exhibit any withdrawal reflexes. Moreover, the percentage of “responders” in TW2 was lower in NDC (28%) as compared to SCI (74%; *p* < 0.05). For obvious reasons, comparative statistical analyses between groups were only feasible for TW2. Following the determination of “responders,” response rates to the 60 stimuli were calculated separately for both study groups and the three stimulation sites. Table 2 summarizes the response rates of individuals with SCI and NDC regardless of the recording site. As already explained above, statistical analysis had to be restricted to TW2. In accordance with the hypothesis, the overall rate of motor responses to 60 stimuli was significantly smaller in NDC than in SCI, irrespective of the stimulation site (Wilcoxon rank-sum test *p* < 0.05, see Table 2 for all stimulation sites).

**TABLE 2:** Response rate (%) of SCI-patients and NDC’s to 60 laser stimuli.

		TW1		TW2			
Site	%	SCI	NDC	SCI	NDC	*p*	*r*
FS	Mean	9.6	0	19.9	2.5	0.02	0.46
	Median	6.7	0	10.0	0		
	Q1; Q3	0.8–11.7	0	0–35.0	0–2.1		
	IQR	10.9	0	35.0	2.1		
FD	Mean	14.4	0	20.7	4.3	0.01	0.49
	Median	10.8	0	6.6	0		
	Q1; Q3	1.3–22.5	0	31.7–38.3	0–1.7		
	IQR	21.2	0	6.6	1.7		
FH	Mean	14.3	0	16.2	3.1	0.02	0.50
	Median	10.8	0	6.7	0		
	Q1; Q3	2.1–20.0	0	0.4–20.4	0.0–2.1		
	IQR	17.9	0	20.0	2.1		

Comparison (Wilcoxon rank-sum test) between the percentage of responses to 60 laser stimuli in SCI and NDC, which includes all recorded reflexes, regardless of whether the tibialis anterior, soleus, or both showed activity. Due to absent reflexes in TW1, statistics are not available. The response rate (%) to laser stimuli in TW2 was significantly higher in SCI as compared to NDC. Effect-size: *r* < 0.3: small; *r* < 0.5: medium, *r* > 0.5: large. FS, sole of the foot; FD, dorsum of the foot; FH, head of the fibula; IQR, interquartile range; NDC, non-disabled controls; Q1, 1st quartile; Q3, 3rd quartile; SCI, spinal cord injury; TW, time window.

### 3.2. Temporal pattern of SCI-related nocifensive reflexes

**FIGURE 2:**
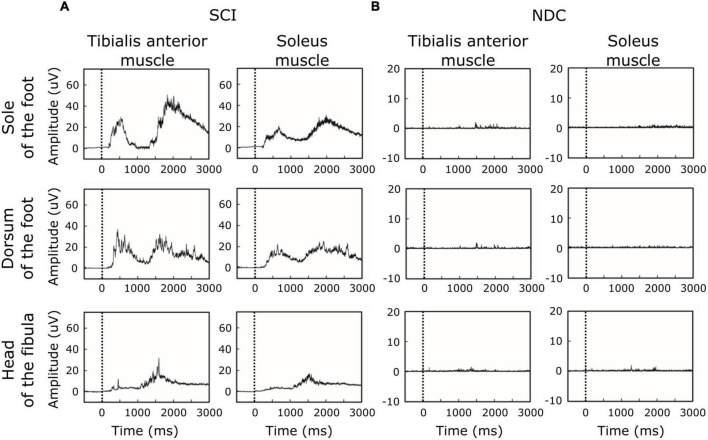
Grand averages of motor responses. **(A)** In participants with spinal cord injury (SCI), averaged EMG-responses to laser stimuli revealed two main time-windows of reflex activity (early ∼250 ms and late > 1,000 ms), suggesting involvement of both A-delta-fibers and C-fibers. In SCI, reflex activity was associated with co-activation of antagonistic muscles (tibialis anterior and soleus muscle). **(B)** In contrast, non-disabled controls (NDC) were characterized by generally less marked muscle responses, only detectable in the late time-window (> 1,000 ms) and without co-activation of the soleus muscle. The black dashed line indicates the onset of the stimulus.

**FIGURE 3:**
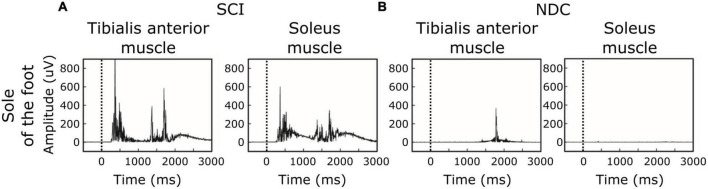
Individual examples of motor responses. **(A,B)** Stimulation site: sole of the foot. **(A)** Ipsilateral EMG-recordings with single reflex events of a participant with spinal cord injury (SCI). **(B)** Corresponding recordings in a non-disabled control (NDC). The black dashed line indicates the onset of the stimulus.

Figure 2 shows averaged motor responses over all participants (grand averages) separated into SCI and NDC. Figure 3 additionally illustrates corresponding individual examples of recorded reflexes. In SCI (Figure 2A) reflex latencies clustered in TW1 and TW2 (Table 3), with the corresponding latencies of TW1 starting around 250 ms (range of mean latency: 187–278 ms, dependent on stimulation site), and TW2 starting at latencies > 1,000 ms (range of mean latency: 1,172–1,460 ms). NDC exhibited flat grand means (Figure 2B) based on only faint motor responses (Figure 3B).

**TABLE 3:** Latencies of laser-evoked withdrawal reflexes.

		TW1			TW2					
Site		SCI		NDC	SCI		NDC			
	ms	TA	SO		TA	SO	TA	SO	*p* (TA)	*p* (SO)
FS	Mean	187	193	n.a.	1,460	1,472	1,571	1,393	0.371	0.564
	± SD	39	40	n.a.	175	189	153	172		
	SEM	16	17	n.a.	66	77	89	100		
FD	Mean	245	259	n.a.	1,172	1,180	1,231	1,318	0.753	0.692
	± SD	81	71	n.a.	254	256	264	196		
	SEM	31	29	n.a.	104	104	152	56		
FH	Mean	278	287	n.a.	1,257	1,230	1,030	1,115	0.534	0.174
	± SD	111	127	n.a.	158	214	249	211		
	SEM	50	63	n.a.	91	151	102	70		

Non-disabled controls (NDC) showed motor responses only in TW2. p-values refer to comparisons between SCI and NDC in TW2, separated for the two muscles (tibialis anterior and soleus) at the three different recording sites. FS, sole of the foot; FD, dorsum of the foot; FH, head of the fibula; NDC, non-disabled controls; SCI, spinal cord injury; SO, soleus muscle; TA, tibialis anterior muscle; TW, time window.

Of note, reflex activity in SCI included simultaneous ipsilateral reflexes in both the TA and SO (see Figures 2,3), indicating co-activation of the two antagonistic muscles. NDC did not show co-activation of both muscles (Figure 2B). Their motor responses were predominantly (78%) recorded from the TA, indicating dominating flexor activity.

Reflex latencies between SCI and NDC were similar in TW2, irrespective of the stimulation site (FS/FD/FH) and localization of recording (TA/SO; Table 3). In TW1, no motor responses were detectable at all in NDC.

**FIGURE 4:**
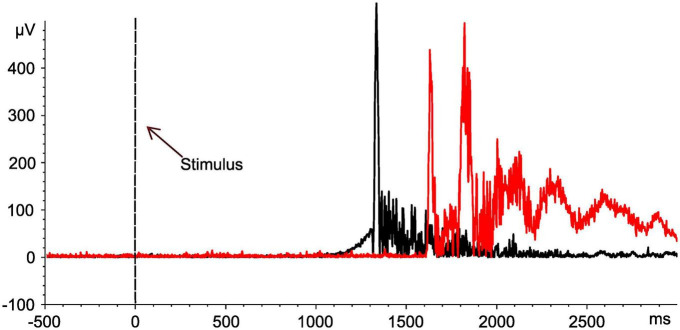
Assessment of nerve conduction velocities for determination of nerve fiber types. Motor responses of an individual with spinal cord injury (No. 10). EMG signals of the tibialis anterior muscle after stimulation at both the head of the fibula (black curve) and the sole of the foot (red curve). Based on the distance between the two stimulation sites (42 cm), the determined shift in latency (561 ms) is compatible with the conduction velocity of the C-fibers (0.75 m/sec). The black dashed line indicates the onset of the stimulus.

As soon as the previously unexpected proof of two distinct TW reproducibly emerged in SCI, the conduction velocity of reflex activity was calculated in a subset of study participants regarding FH-related reflexes (SCI: 10/15; NDC: 8/12) to confirm the role of C-fibers for the responses in TW2 based on the following approach: (1) the latency difference between the activity in TW2 recorded after stimulating the FH and the FS and (2) the individual distance between the two stimulation sites. These measurements were performed at the end of the experiment (see Figure 4 for an individual example). The calculated conduction velocity was well in line with knowledge on the conduction velocity of C-fibers and did not differ between the two study groups (SCI: mean 1.34 ± 0.46 m/sec; NDC: mean 1.08 ± 0.38 m/sec; *p* > 0.05) (Treede et al., 1995). The conduction velocity of TW1 ranged between 5.2 and 16.7 m/sec, which corresponds well with A-delta fiber activity.

### 3.3. Large response magnitude of nocifensive reflexes in SCI

**FIGURE 5:**
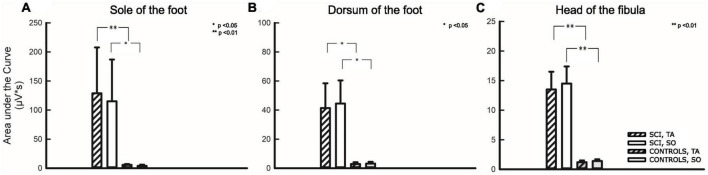
Area under the curve analysis for three stimulation sites. Stimulation sites: **(A)** Sole of the foot, **(B)** Dorsum of the foot, **(C)** Head of the fibula. The first two bars on the left relate to participants with SCI, the two following bars relate to non-disabled controls. Hatched bars depict the area under the curve analysis of the tibialis anterior (TA), while uniformly filled bars depict the area under the curve analysis of the soleus (SO) muscle. Asterisks reflect significant differences in comparison between individuals with and without SCI at the respective sites and corresponding muscles. *p < 0.05; **p < 0.01.

Beyond evaluating the percentage of motor responses and their latencies, the magnitude of the respective reflex activity (AUC) was assessed. Results are given in Table 4 and Figure 5.

**TABLE 4:** Area under the curve.

		TW 1				TW 2							
Site		SCI			NDC	SCI			NDC				
		TA	SO	#*p*		TA	SO	#*p*	TA	SO	#*p*	†*p* (TA)	†*p* (SO)
FS	Mean	29.7	25.6	0.485	n.a.	128.8	115.1	0.945	5.7	4.0	0.500	0.006	0.024
	± SD	27.3	31.1		n.a.	209.0	175.7		2.9	3.6			
	SEM	11.1	31.1		n.a.	79.0	71.8		1.4	2.1			
	Max	67.3	84.1		n.a.	592.8	468.7		8.5	8.0			
	Min	4.7	3.6		n.a.	13.0	12.7		1.9	1.0			
FD	Mean	50.3	39.9	0.808	n.a.	41.4	44.5	0.867	2.9	3.2	0.955	0.026	0.020
	± SD	85.3	60.6		n.a.	48.1	42.1		2.9	3.4			
	SEM	32.3	24.7		n.a.	17.0	15.9		1.1	1.2			
	Max	236.6	159.5		n.a.	120.4	105.1		7.4	9.0			
	Min	1.3	1.3		n.a.	1.4	2.9		0.5	0.3			
FH	Mean	16.1	17.7	0.911	n.a.	13.5	14.5	0.262	1.2	1.4	0.166	0.006	0.004
	± SD	21.1	19.9		n.a.	7.3	6.5		0.8	0.5			
	SEM	9.4	9.9		n.a.	3.0	2.9		0.3	0.3			
	Max	53.0	47.2		n.a.	21.4	20.1		2.3	2.0			
	Min	1.6	5.4		n.a.	1.6	7.8		0.2	0.9			

Area under the curve (AUC) in SCI and NDC (compared with each other for the respective time-windows and recording sites). #*p*: AUC of tibialis anterior muscle compared with AUC of soleus muscle; †*p*: comparison between SCI and NDC (TW2 only). FS, sole of the foot; FD, dorsum of the foot; FH, head of the fibula; NDC, non-disabled controls; SCI, spinal cord injury; SO, soleus muscle; TA, tibialis anterior muscle; TW, time window.

As already described above, a comparison of AUC between the two groups (SCI/NDC) was restricted to TW2 and required that individuals among NDC showed motor responses at all. A quantitative difference of approximately two orders of magnitude was found between AUC in favor of individuals with SCI—TA: FS *p* = 0.006; FD *p* = 0.026; FH *p* = 0.006; SO: FS *p* = 0.024, FD *p* = 0.020, FH *p* = 0.004.

### 3.4. Pain ratings to noxious radiant heat stimuli are dependent on the severity of SCI

The rating of laser stimuli (NRS 0–100) was lower (*p* < 0.001) in SCI as compared to NDC (FS_*SCI*_: 4.2 ± 7.3; FS_*NDC*_: 14.6 ± 8.9; FD_*SCI*_: 4.5 ± 6.7; FD_*NDC*_: 15.7 ± 11.0; FH_*SCI*_: 1.7 ± 2.7; FH_*NDC*_: 14.4 ± 9.1). A considerable proportion in SCI did not sense any heat stimuli at all (percentage of analgesia: FS: 46%, FD: 40%, FH: 50%). As expected, no participant with sensorimotor complete SCI (AIS-A) reported any painful sensation with respect to the applied laser stimuli. Correlation analyses indicated no significant correlation between the perceived pain intensity and the motor response rates (*p* > 0.05). Similarly, no association was found between the perception of noxious laser-ratings, as rated by NRS, and the categories responder/non-responder (*p* > 0.05) as well as neuropathic/no neuropathic pain (Eta-coefficient 0.07, *p* > 0.05).

### 3.5. Interrelation between reflex activity, spasticity, and neuropathic pain perception

Referring to spasticity and laser-evoked reflex behavior, an association between the percentage of motor responses and the two spasticity scores “SCAT” and “MAS” was found. Accordingly, the percentage of withdrawal reflexes in SCI correlated positively both with the MAS (FD, FS, FH) and the SCAT (FD, FH, Kendall-tau-b *p* < 0.05, see Table 5).

**TABLE 5:** Relation between reflex response rates and clinical assessments.

	Assessment							
Site	SCAT		MAS		Numeric rating scale		CPG (yes/no)	
	Kendall’sτ	*p*	Kendall’sτ	*p*	Kendall’s τ	*p*	η	*p*
FS	0.262	0.117	0.363	**0.047**	0.026	0.922	0.164	0.592
FD	0.378	**0.031**	0.426	**0.016**	−0.098	0.635	0.356	0.193
FH	0.424	**0.050**	0.506	**0.023**	0.361	0.106	0.118	0.745

Bold values represent the significant results (*p* < 0.05). CPG, clinical pain grading; η, eta-coefficient; FS, sole of the foot; FD, dorsum of the foot; FH, head of the fibula; MAS, Modified Ashworth Scale; SCAT, spinal cord assessment tool for spastic reflexes.

Central neuropathic pain and spasticity were found to be inversely associated (Fisher’s exact test *p* = 0.04, Φ = 0.61), with only 25% (2/8) of individuals with central neuropathic pain presenting spasticity. In contrast, 86% (6/7) of SCI-individuals without central neuropathic pain were characterized by relevant spasticity (MAS or SCATS > 1 in any joint examined; also see Table 1). Accordingly, there was also an association between central neuropathic pain presentation (CPG = yes) and severity of spasticity (Eta-coeffcient_*MAS*_ 0.59, *p* = 0.04; Eta-coeffcient_*SCAT*_ 0.69, *p* < 0.001). Further analyzes testing for a correlation of spontaneous pain severity (NRS 0–10) and spasticity implied a negative, albeit insignificant association (*p* > 0.05). No association was either found between spontaneous pain severity and the percentage of motor responses (*p* > 0.05, Table 5). Similarly, the presence of neuropathic pain did not seem to have an impact on the reflex behavior in the group of responders with SCI (*p* > 0.05, Table 5).

## 4. Discussion

This study was the first to apply radiant heat laser stimuli to investigate nociceptive withdrawal reflexes in individuals with SCI compared with non-disabled controls. It provided insights proving the involvement of both A-delta- and C-fibers in the mediation of nocifensive reflexes in SCI. As expected, hyperreflexia was found in SCI and confirmed to be associated with spasticity as a widely accepted clinical presentation of spinal hyperexcitability. Yet, contrary to our expectation, we found no association between the reflex behavior and the presence/absence of central neuropathic pain, albeit the latter being also regarded to be a consequence of spinal hyperexcitability or disinhibition. The present findings may imply that although nocifensive hyperreflexia—as assessed in this study—may be mediated by the same spinal (interneuronal) circuitries as spasticity, central neuropathic pain may be mediated differently. A potential explanation in this context could be disparate mechanisms of maladaptive plasticity being involved in the development of either complication of SCI.

### 4.1. Hyperexcitability in spinal cord injury underlies characteristic nocifensive reflex behavior

As equivalent of prevalent hyperresponsiveness, laser-evoked withdrawal reflexes have more frequently been found in SCI than in NDC, with more than two-thirds in the SCI-cohort displaying reflexes that could be elicited at a mean rate of approximately 20% and responses two orders of magnitude higher. As opposed to SCI, detectable motor responses in NDC only occurred in a C-fiber- but not in a A-delta-fiber related time window. In SCI, a similar bi-component reflex behavior within a comparable time-range had been reported in 1948 by application of the heated shaft of a reflex hammer to elicit nociceptive reflexes (Kugelberg et al., 1960). By means of a selective mechanical blocking of A-delta fibers, it could be demonstrated that both A-delta and C-fiber-afferents contribute to the composite reflex behavior. Although a direct method of A-fiber blocking was not performed in the present study, the conduction velocities could be estimated. The results thus obtained indicated that the responses in both time windows were also in well-accordance with the engagement of A-delta-fibers and C-fibers, respectively (Willer et al., 1979; Treede et al., 1995; Mørch et al., 2007). Systematic studies on the selective contribution of C-fibers to nociceptive withdrawal reflexes are rare, essentially based on animal studies and challenging with respect to the appropriate stimulation protocol (Schomburg et al., 2000; Kimura et al., 2004; Kimura and Kontani, 2008; Hsieh et al., 2015). Simultaneous recording of A-delta and C-fiber-related reflexes under healthy conditions is even more challenging, since the two fiber types differ with respect to their optimal recruitment/activation thresholds. Appropriate stimulation paradigms which are capable of simultaneously eliciting two-component withdrawal reflexes have mostly been used in animal experiments to investigate fiber-related differential effects of drugs (Kimura et al., 2004) and to explore pathomechanisms such as the impact of wind-up on the A-delta- and C-fiber related reflex component (Kimura et al., 2004; Kimura and Kontani, 2008). The peculiarity of the present stimulation paradigm, which only resulted in bi-component reflexes in SCI and not in NDC may thus be of particular value to selectively clarify the contribution of A-delta and C-fiber afferents in conditions of spinal pathology and, even more, may be a useful tool for future pharmacological trials in this field.

In non-disabled control subjects, the absence of reflex activity in the A-delta-related time window is currently hardly explained. The different classes of thermoreceptors for noxious heat operate over a different temperature range. The temperature range to activate C-fibers in humans is lower (37–49°C) (Treede et al., 1990; Schmidt et al., 1997) than for activating Type 1 A-delta afferents (median threshold > 53°C) (Schepers and Ringkamp, 2009). However, the laser energy used was not expected to have preferentially activated C-fibers. In fact, similar laser energies have been used in other studies and showed robust results indicative of recruitment of A-delta fibers (Schuh-Hofer et al., 2015). An alternative explanation for the pattern of reflex responses in NDC may be volitional movements in TW2. NDC-responders were not systematically asked to judge their reflex behavior as either voluntary or involuntary. Yet, this explanation remains questionable because the latencies of motor responses in NDC did not differ from those observed in C-fiber mediated responses in SCI.

Notably, the reflex behavior of participants with SCI in this study was characterized by concomitant activation of the TA and SO, which may be surprising at first, as withdrawal reflexes are expected to facilitate an effective avoidance reaction to noxious stimuli (Sandrini et al., 2005). However, the present finding is backed by prior studies in individuals with SCI that also reported on simultaneous reflex activity in antagonistic muscle groups (Grimby, 1963; Andersen et al., 2004), which was explained by expansion and overlap of RRFs for flexor and extensor muscles in SCI. This expansion was to be due to the lack of descending inhibitory control and/or increased sensitivity of the spinal reflex loop in SCI.

### 4.2. Lacking association between hyperreflexia and central neuropathic pain: an indication of separate maladaptive circuits?

Provided that the spinal cord and not the cauda equina is affected, hyperreflexia is known to prevail in individuals with (traumatic) SCI and is widely accepted to be the result of an imbalance between excitatory and inhibitory neural input (Hiersemenzel et al., 2000; Sangari et al., 2021), after an initial phase of spinal shock (Atkinson and Atkinson, 1996). This could now also be shown based on hyperresponsiveness to nociceptive withdrawal reflexes in chronic SCI triggered by noxious laser stimuli. Together with hyperreflexia, spasticity is a further common phenomenon following the injury of central motor pathways and was indeed positively associated with the reflex behavior in the cohort investigated (Dietz and Sinkjaer, 2012). This finding is likely to reflect enhanced interneuronal excitability due to SCI, leading to a decreasing threshold and increasing magnitude of reflex behavior over time (Hiersemenzel et al., 2000; Elbasiouny et al., 2010). Neuropathic pain is also discussed to be a phenomenon that is, on the one hand, maintained by pathomechanisms such as an impaired modulation of inhibition or sensitization of neuronal activity—leading to hyperexcitability as “driving force”—and, on the other hand, assumed to be influenced by individual processes of plasticity (Nielsen et al., 2007; Zeilig et al., 2012; Corleto et al., 2015; Nees et al., 2016; Colloca et al., 2017; Finnerup, 2017; Sliwinski et al., 2018). Here, no association has been found between nocifensive reflex behavior—as characterized by hyperreflexia—and central neuropathic pain. Moreover, indications emerged suggesting that central neuropathic pain and spasticity may even be inversely associated. Given this discrepancy, the question of underlying causes arises. Particularly since existing studies show a positive association between spasticity and pain perception (McKay et al., 2018). However, it should be noted here that spasticity is indeed known to trigger pain, although not neuropathic pain as specifically investigated in this study, but nociceptive and musculoskeletal pain (Bryce et al., 2012b). Now, considering that studies of spasticity in SCI often focus rather non-specifically on pain and do not attempt to precisely differentiate the underlying pain entity, the mentioned discrepancy between pain and spasticity in this compared with other studies can be explained (Shaikh et al., 2016). However, notwithstanding the already discussed relationships between hyperexcitability, hyperreflexia, and spasticity, little is known factually about how structural changes could be capable of functionally modifying reflex circuits. It is assumed that at least some degree of descending motor connectivity must be intact for spasticity to occur and that a certain pattern of ascending pathway lesion (lemniscal versus spinothalamic tract) is required to pave the way for central neuropathic pain to develop (Finnerup et al., 2003; Levitan et al., 2015; Finnerup, 2017; Sangari et al., 2019). In consequence of these aspects, it may be conceivable that spasticity and hyperreflexia are associated because possible SCI-related structural or functional changes (maladaptive plasticity) are spinally cross-linked to an efferent pathway (lower motoneurons). In contrast, the emergence of neuropathic pain is known to be not only the consequence of structural and functional changes at spinal cord level, but to be finally modulated by a highly complex supraspinal pain network. Our failure to show an association between the reflex behavior and neuropathic pain may thus be at least partially explained by a strong contribution of top-down pain modulating factors (Kuner and Flor, 2016). From this perspective, our finding may thus exemplify how pathologic nociceptive processing at spinal cord level, manifested by pathologic reflex behavior, is dissociated from the conscious perception of pain (Wasner et al., 2008; Costigan et al., 2009; Cruz-Almeida et al., 2012; Colloca et al., 2017; Huynh et al., 2021). Taken together, central neuropathic pain after SCI remains to be most likely based on multiple factors. Some of them paving the way toward its emergence, others facilitating its clinical occurrence and individual severity. In this context, the presented findings provide further indications that suggest different types of maladaptive plasticity at the spinal level or beyond as partial explanation for the two differently related clinical sequalae of SCI, investigated in this study: spasticity and central neuropathic pain. Nevertheless, the low sample size must be carefully considered with respect to subgroup analyses when drawing such conclusions, still emphasizing the need of further focused research to underpin the present findings. In addition, since a substantial proportion of our study participants took either anticonvulsants or baclofen, we cannot exclude that these central nervous system drugs have biased the presented results. Accordingly, it remains eventually unclear whether the inverse relationship found between spasticity and spontaneous neuropathic pain is indeed a robust reflection of disparate mechanisms of plasticity in the sensory and motor system after SCI or rather influenced by an underlying confounding factor such as a selection bias related to the small sample size.

## 5. Study limitations

This proof-of-principle study focused on investigating the presumed differences between reflex responses to laser (radiant heat) stimuli in individuals with SCI and NDC. Consequently, the presented study was not primarily designed to examine specific differences in reflex behavior with respect to stimulation or recording sites. However, this could have provided further insight into the modular reflex organization of laser-evoked motor responses in individuals with SCI. To further address this question, future studies will involve a larger number of antagonistic muscle groups. In contrast to previous studies (Andersen et al., 2004; Biurrun Manresa et al., 2013, 2014) the RRFs of the study participants were also not investigated. Thus, the question of possible enlargement of the RRF in SCI was not addressed. Given that the enlargement of RRF—like hyperreflexia—is assumed to reflect pathomechanisms of central sensitization, we propose to include this valuable outcome parameter in future studies of laser-evoked motor responses in individuals with SCI.

## 6. Conclusion and outlook

While spasticity and neuropathic pain arguably underlie general spinal hyperexcitability or disinhibition after SCI, there is emerging evidence that distinct types of maladaptive plasticity may be responsible for the clinical development of spasticity and neuropathic pain. Thus, laser-evoked nocifensive reflexes assessed with the presented stimulation paradigm may represent a promising yet unique assessment to evaluate nociceptive processing after human SCI. Such a technique may, for instance, be suitable to specifically investigate the involvement of A-delta fibers and C-fibers in clinical studies, e.g., targeting novel drug therapies or spinal maladaptive neuroplastic changes (Clarke et al., 2002; Lo et al., 2004). To verify the assumption of inversely related spasticity and neuropathic pain, a further step would be to initiate targeted, ideally translational, research efforts to explicitly evaluate the excitation level of the nervous system and the structural lesion pattern of the spinal cord in the light of central neuropathic pain presentation.

## Data availability statement

The raw data supporting the conclusions of this article will be made available by the authors, without undue reservation.

## Ethics statement

The studies involving human participants were reviewed and approved by the Ethics Committees of the Medical Faculties of Heidelberg and Mannheim (S-660/2013 and 603N/2014), Heidelberg University, Germany. The patients/participants provided their written informed consent to participate in this study.

## Author contributions

NW, R-DT, SF, and SS-H designed the study and interpreted the results. AT-T, LH, and SS-H performed the research. SF and SS-H analyzed data and wrote the manuscript. LH drafted the figures. All authors contributed to the article and approved the submitted version.

## Footnotes

1
https://www.emsci.org
